# Whole genome sequence-based characterization of virulence and antimicrobial resistance gene profiles of *Staphylococcus aureus* isolated from food poisoning incidents in eastern China

**DOI:** 10.3389/fmicb.2023.1225472

**Published:** 2023-09-19

**Authors:** Shuyang Yu, Yuxuan Zhou, Dan Feng, Quangui Jiang, Tianle Li, Guilai Jiang, Zhemin Zhou, Heng Li

**Affiliations:** Pasteurien College, Suzhou Medical College of Soochow University, Suzhou, China

**Keywords:** *Staphylococcus aureus*, food poison, diarrhea, antimicrobial resistance, virulence

## Abstract

*Staphylococcus aureus* is an opportunistic foodborne pathogen occasionally isolated from diarrhea patients. In recent years, increasing studies have reported the detection of *S. aureus* in food poisoning incidents due to food contamination in the North and South of China. However, the epidemiology and genetic characteristics of *S. aureus* from food poisoning incidents in Eastern China remain unknown. The present study examined the genetic characteristics, antimicrobial resistance, and virulent factors of multidrug-resistant *S. aureus* isolated from 22 food poisoning incidents reported by the hospitals and health centers in Eastern China from 2011 to 2021. A total of 117 resistant and enterotoxigenic *S. aureus* isolates were collected and sequenced, among which 20 isolates were identified as methicillin resistant. Genetic analysis revealed 19 distinct CC/ST types, with CC6, CC22, CC59, CC88, and CC398 being the most frequent variants in methicillin-resistant *S. aureus* (MRSA). A considerable shift in CC types from CC1 to CC398 between 2011 and 2021 was observed in this study, indicating that CC398 may be the main epidemic strain circulating in the current food poisoning incidents. Additionally, genes for enterotoxins were detected in 55 isolates, with a prevalence of 27.8% (27/97) for methicillin-sensitive variants and 35.0% (7/20) for MRSA. The *scn* gene was detected in 59.0% of the isolates, demonstrating diverse contaminations of *S. aureus* among livestock-to-human transmission. Of the 117 isolates, only ten isolates displayed multi-drug resistance (MDR) to penicillin, tetracycline, and macrolides. None of the 117 foodborne *S. aureus* isolates tested positive for *vanA* in this study. Together, the present study provided phylogenetic characteristics of *S. aureus* from food poisoning incidents that emerged in Eastern China from 2011 to 2021. Our results suggested that these diarrhea episodes were hypotonic and merely transient low-MDR infections, however, further research for continued surveillance given the detection of virulence and antimicrobial resistance determinants is required to elucidate the genomic characteristics of pathogenic *S. aureus* in food poisoning incidents in the context of public health.

## Introduction

*Staphylococcus aureus* is an opportunistic pathogen frequently detected in food as well as on the skin, nose and throat of humans (Argudín et al., 2010). It can cause acute food poisoning, which presents symptoms such as diarrhea, stomach pain, nausea, vomiting, and other related manifestations (Johler et al., 2015; Mahros et al., 2021). From 2005 to 2018, the European Centre for Disease Prevention and Control (ECDC) documented over 500,000 *S. aureus* bloodstream infections in European countries (Gagliotti et al., 2021). Similarly, over 241,000 illnesses of foodborne *S. aureus* infection were reported annually by the Centers for Disease Control and Prevention (CDC) in the United States (Kadariya et al., 2014). Previous research in China revealed frequent detection of *S. aureus* in animal meat, egg products, and dairy products, with a positive rate of 35.0% (Wu et al., 2018). In addition, a total of 1,150 *S. aureus* were isolated from 24 provinces in China, with 4.3% of retail foods being contaminated by *S. aureus*, and 7.9% of retail foods isolates being *mecA* positive. Moreover, 97.6% of *S. aureus* isolates were resistant to at least one antimicrobial compound, and 57.5% of these were multi drug resistant to penicillin (83.7%), linezolid (67.7%) and erythromycin (52.1%) (Wang et al., 2017).

Foodborne *S. aureus* is associated with various virulent factors, including genes responsible for producing enterotoxins, exfoliative toxins, toxic shock syndrome toxin (*tsst*-1), Panton-Valentine leucocidin, staphylococcal complement inhibitor, and hemolysins (Leung et al., 1993; Gouaux et al., 1997; Bhatia and Zahoor, 2007; Boyle-Vavra and Daum, 2007; Bukowski et al., 2010; Carrera et al., 2017; de Jong et al., 2018). Among these factors, enterotoxins are considered the primary culprits in causing staphylococcal food poisoning (SFP) (Bhatia and Zahoor, 2007). According to a recent study, Guo et al. reported an SFP outbreak caused by ST7 *S. aureus* strains in two kindergarten campuses in South China (Guo et al., 2023). Six antimicrobial resistance genes were detected including *blaZ*, *ANT (4′)-Ib*, *tetK*, *lnuA*, *norA*, and *lmrS*. Another study in South China analyzed the clonal complex (CC) of 62 distinct *S. aureus* strains and found that CC239 and CC3 were the dominant clones of food poisoning incidents (Xie et al., 2016). SCCmecIII-ST239 was the prevalent type, accounting for 43.4 to 79.5% of hospital and community-associated MRSA and harboring a series of virulence genes such as *sea, seb, seh, eta,* and *pvl* (Xie et al., 2016). Additionally, a total of 138 foodborne *S. aureus* were isolated from outbreaks in North China, with CC1, CC5, CC7, CC8, CC15, CC59, CC88, CC97, and CC398 as the predominant clones (Lv et al., 2021). However, there is a lack of research investigating the genetic characteristics of foodborne *S. aureus* in Eastern China during the past decade.

To identify *S. aureus* strains isolated from food poisoning incidents in Eastern China from 2011 to 2021, a combined bioinformatics approach was employed to study the genetic characteristics. In addition, the genotypic antimicrobial resistance profiles and virulent factor possession were examined for a comprehensive assessment of the potential risks associated with the presence of methicillin resistance in food poisoning incidents.

## Materials and methods

### Ethics approval

The experiment was strictly conducted according to the Guide for Care and Use from the Research Ethics Committee of Soochow University (20210220). All procedures involving human participants were performed to the ethical standards. Patients were given informed consent to participate in the study.

### Description of food poisoning incidents

Information on the food poisoning incidents was gathered from the Center for Disease Control and Prevention of Suzhou, China. A total of 896 stool samples (n = 850) and vomit samples (n = 46) from diarrheal patients were collected from 22 hospitals and community health centers between 2011 and 2021 and forwarded for testing using the Chinese National Foodborne Disease Surveillance Manual (Li et al., 2018). These 22 facilities were dispersed among ten regions, between 30°93’N to 35°55’N and 120°21′E to 121°64′E in Eastern China. The collected samples were tested for pathogenic bacteria including *Salmonella*, *Shigella*, *Staphylococcus aureus*, *Bacillus cereus*, *Vibrio parahaemolyticus*, diarrhea-causing *Escherichia coli*, and *Listeria monocytogenes*. Food poisoning incidents with *Staphylococcus aureus* as the dominant pathogen were enrolled for further analysis. Detail descriptions of CC, ST types, isolation of year and geographic data of *S. aureus* were shown in Supplementary Table S1.

### Bacterial isolation and identification

The present research was conducted during the period from 2011 to 2021. In the initial step, 10 g of stool samples and vomit samples were collected and transferred to the laboratory on ice. Then samples were homogenized in 0.1% peptone saline in a filter bag (Bkmam, Changde, China). After that, 100 ul were cultured onto Baird Parker agar (HopeBio 4,115, Beijing, China) and CHROMagar™ MRSA agar (Becton Dickinson, Franklin Lakes, NJ). The plates were incubated in the carbon dioxide incubator overnight at 37°C. Then, a loop full of bacterial culture from incubated tubes was streaked separately into the Baird Parker agar, and the plates were examined and studied carefully for the presence of characteristic colonies of *S. aureus*.

The *S. aureus* strains were identified by 16 s rRNA sequencing and MALDI-TOF MS (Bio-Merieux, Craponne, France). Generally, a 1.4-kb fragment of the 16S rRNA gene was amplified by PCR with universal primers (27F, 5’-AGAGTTTGATCMTGGCTCAG-3′, 1492R, 5’-TACGGYTACCTTGTTACGACTT-3′). The PCR product was purified and sequenced in both directions by use of conserved-region primers on the platform of Honsunbio company (Shanghai, China). Purified sequencing results were processed and edited by ABI 3100 (Applied Biosystems) and Sequencher (Gene Codes, Ann Arbor, Mich.), respectively. The altered sequences were identified by comparing them to GenBank via Blast. In the case of bacteria with a low identity, MALDI-TOF MS was enrolled for detection. Briefly, each sample was inoculated as triplicates on the target plate and covered by the freshly prepared matrix (Bruker Daltonik GmbH, Bremen, Germany). Mass spectra were compared with spectra obtained from the associated database (Hansen and Lee, 2017; Holzknecht et al., 2018).

### Antimicrobial susceptibility testing

Susceptibility testing of confirmed *S. aureus* strains was performed according to the Clinical and Laboratory Standards Institute disk diffusion method (CLSI 2022). *Staphylococcus aureus* ATCC 25923 was used as a control strain. Disks from Oxoid were used. The following antimicrobial disks were tested: ampicillin (10 μg), penicillin (10 units), cefoxitin (30 μg), ceftazidime (30 μg), chloramphenicol (30 μg), clindamycin (2 μg), erythromycin (15 μg), gentamicin (10 μg), linezolid (30 μg), and tetracycline (30 μg). Cefoxitin was tested as a surrogate marker for the detection of methicillin resistance.

### Whole genome sequencing

After growing the isolates for 24 h at 37°C in Tryptone Soya Broth (AOBOX 02–049, Beijing, China), genomic DNA was extracted and purified using a HiPure Bacterial DNA Kit (D3146, Meiji Biotechnology Co., Ltd., Guangzhou, China). Library construction was performed with Vazyme TruePrep DNA Library Prep Kit TD501 (Vazyme, Nanjing, China). A Nanodrop ND-1000 spectrophotometer (Nanodrop Technologies, Wilmington, DE, United States) and a 1.0% (w/v) agarose gel were used to assess the quality of the extracted DNA. Purified DNA was whole-genome sequenced using Illumina HiSeq 4,000 platform (150 bp paired-end reads with ~200-fold average coverage) on the Honsunbio platform (Shanghai, China). The raw reads were trimmed, and genome was assembled by EToKi v1.0 (Zhou et al., 2020). The QUAST v2.3 was utilized to assess the quality of the genome assembly (Gurevich et al., 2013). The raw sequencing reads were uploaded to the China National GenBank with the accession number of CRA010922. The sequences can be accessed at https://bigd.big.ac.cn/gsa/browse/CRA010922.

### Bioinformatics analysis

To classify STs into clonal complexes (CCs), the Illumina read files were subjected to multilocus sequence typing (MLST.20) and eBURST v3 analysis (Urwin and Maiden, 2003; Feil et al., 2004). A maximum likelihood core-genome phylogenetic tree was constructed using 117 strains based on 547 core genes (~96,496 SNPs). The *S. aureus* ATCC 25923 was employed as the reference genome for SNP analysis. The select minimum depth at SNP positions was set to 10x, while select minimum relative depth at SNP positions was set to 10%. The select minimum distance between SNPs (prune) was set to 10 bp and select minimum SNP quality was set to 30. The select minimum read mapping quality was set to 25 and the select minimum Z-score was set to 1.96 in CSI Phylogeny v1.4 (Kaas et al., 2014).

Antimicrobial-resistant genes and virulent factors were determined using the ResFinder, Mobile ElementFinder, and VFDB databases, with a minimum of 60% nucleotide identity retained in all algorithms (Liu et al., 2019; Bortolaia et al., 2020; Johansson et al., 2021). The detection of *arg, icaA, icaB, icaC, icaD, icaR, luxS,* ΦSa3 and ΦAVβ (SAAV_2008, SAAV_2009) genes was blast in MyDbFinder 2.0 with the select threshold of 98% and select minimum length of 60% on the platform of Centre for Genomic Epidemiology.^1^

### Statistical analysis and visualization

The line chart and donut chart were made using GraphPad Prism 7, and statistical significance was assessed using One-way ANOVA with *p* < 0.05. The genotypic data were visualized in Grapetree and iTOL (Urwin and Maiden, 2003; Letunic and Bork, 2019).

## Results and discussion

### Description of the food poisoning incidents in eastern China from 2011 to 2021

**Figure 1:**
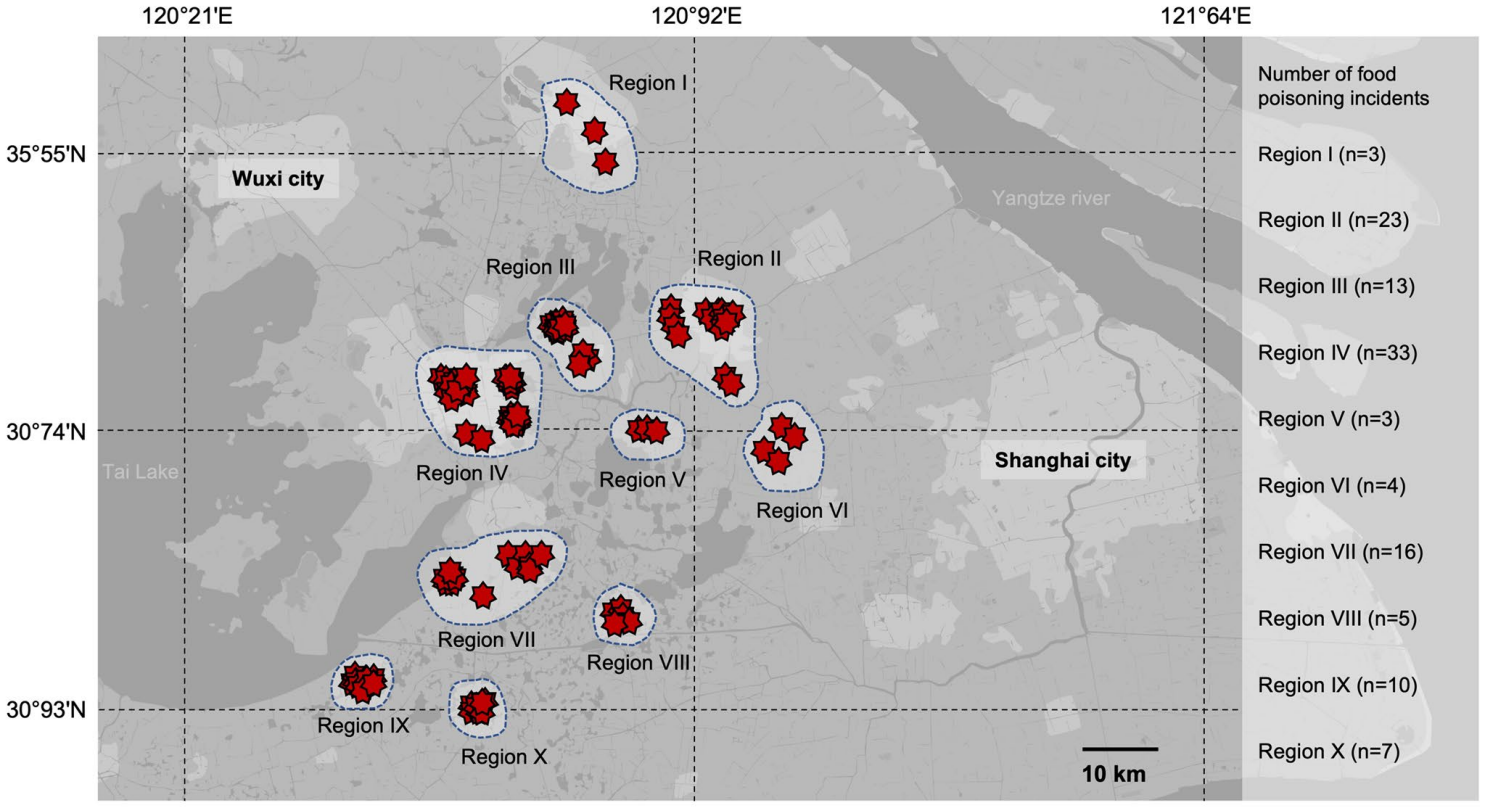
Geographic distribution of food poisoning incidents in Eastern China from 2011 to 2021. The food poisoning incidents collected for this study were distributed between 30°93’N and 35°55’N and 120°21′E to 121°64′E. A total of 117 strains of *S. aureus* were isolated and distributed to ten regions. The red stars represent the outbreaks of each food poisoning incident in the region.

In this investigation, the food poisoning incidents collected for this study were distributed between 30°93’N and 35°55’N and 120°21′E to 121°64′E (Figure 1). A total of 117 strains of *S. aureus* were isolated from 896 stool and vomit samples collected from diarrheal patients who registered to the 22 hospitals and community health centers between 2011 and 2021 in Eastern China. Among the 117 isolates, 20 isolates were identified as methicillin-resistant *S. aureus* (MRSA), with the majority of the strains collected from Region IV and Region II. Among them, six strains were isolated from vomit samples and the remaining 111 strains were detected from diarrheal stool. Further epidemiological analysis revealed that the food poisoning incidents in this study were divided sporadically into ten regions, with Suzhou (Region IV) and Kunshan (Region II) being the dominant regions of food poisoning incidents (Figure 1).

### Genetic diversity of *Staphylococcus aureus* isolates from the food poisoning incidents

**Figure 2:**
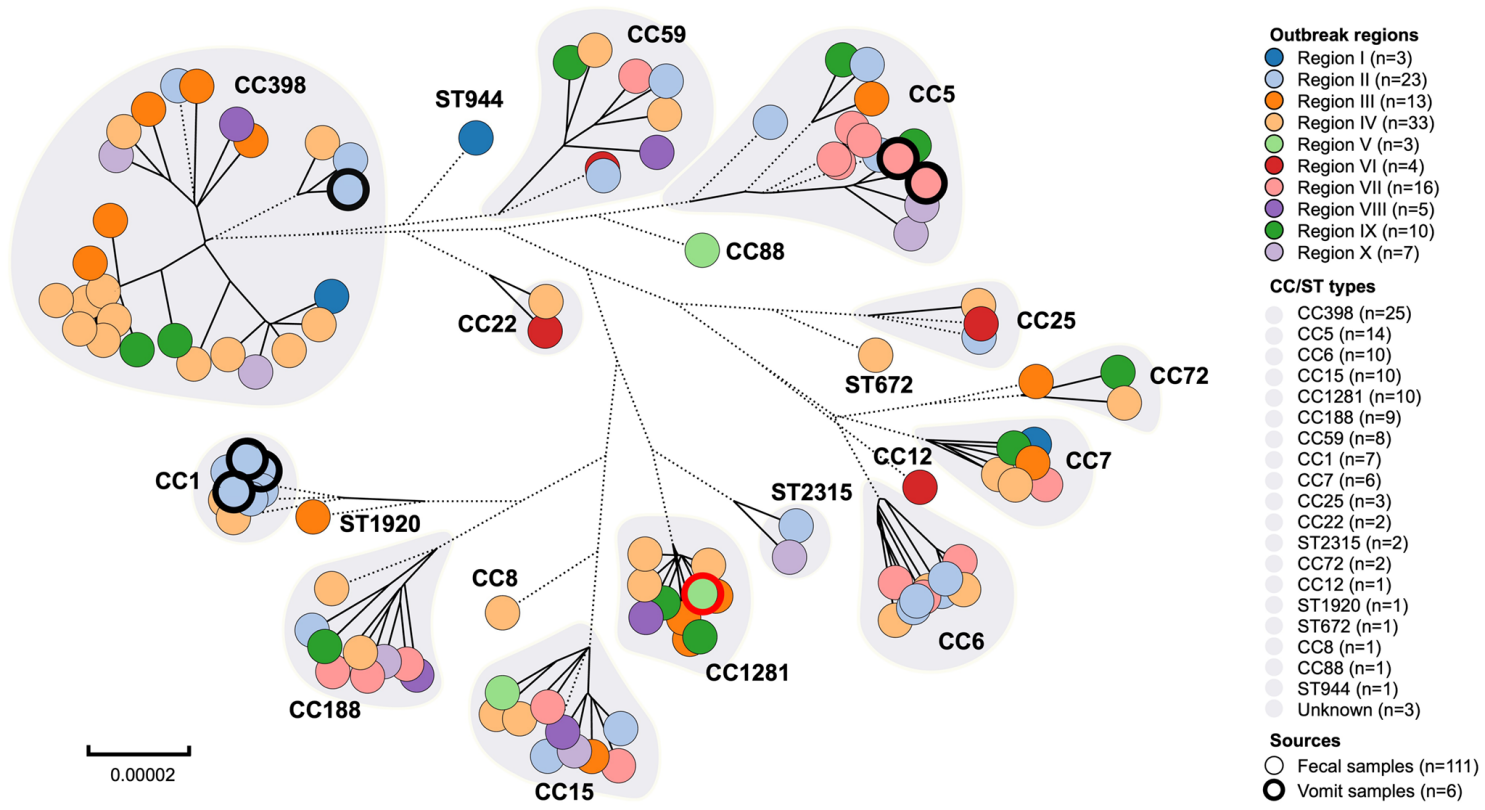
Clonal complexes (CC) and sequence types (ST) of *S. aureus* from food poisoning incidents in Eastern China. The dotted branches represented shorter distances longer than 0.00003, while the solid lines represented the genetic distance between strains.

The MLST typing of the 117 foodborne *S. aureus* genomes identified 19 unique CC/ST types, namely CC1, CC5, CC6, CC7, CC8, CC12, CC15, CC22, CC25, CC59, CC72, CC88, CC188, CC398, CC1281, ST672, ST944, ST1920, and ST2315 (Figure 2, Table 1). MRSA was detected in 20 isolates including CC6, CC22, CC59, CC88, and CC398, while MSSA isolates (n = 97) were widely distributed in the majority of the CC/ST types, indicating the diversity of *S. aureus* contamination in the current food poisoning incidents. Details of the housekeeping genes in MLST typing were described in Supplementary Table S2.

**Table 1:** CC, MRSA/MSSA, ST types and phenotypic antimicrobial resistance of foodborne *S. aureus* isolates from this study.

CC type	MRSA/MSSA	ST^a^	Source^b^	Isolation year^b^	Phenotypic antimicrobial resistance^c^
CC1	0/7	ST1 (7)	Stool (4), Vomit (3)	2011 (1), 2016 (6)	AMP (1), PEN (1)
CC5	0/14	ST5 (14)	Stool (12), Vomit (2)	2017 (2), 2019 (9), 2020 (3)	AMP (7), PEN (7), ERY (9), TET (1)
CC6	2/8	ST6 (10)	Stool (10)	2011 (1), 2019 (4), 2020 (5)	AMP (8), PEN (8), FOX (2), ERY (1), TET (1)
CC7	0/6	ST7 (6)	Stool (6)	2011 (1), 2019 (3), 2020 (1), 2021 (1)	AMP (5), PEN (5), ERY (3), TET (3), GEN (3)
CC8	0/1	ST8 (1)	Stool (1)	2011 (1)	ERY (1)
CC12	0/1	ST12 (1)	Stool (1)	2020 (1)	PEN (1)
CC15	0/10	ST15 (10)	Stool (10)	2011 (1), 2014 (1), 2016 (1), 2019 (5), 2020 (1), 2021 (1)	AMP (9), PEN (9), ERY (1)
CC22	1/1	ST22 (2)	Stool (2)	2019 (1), 2020 (1)	AMP (2), FOX (1), PEN (2)
CC25	0/3	ST25 (3)	Stool (3)	2019 (1), 2020 (2)	ERY (1)
CC59	7/1	ST59 (8)	Stool (8)	2011 (1), 2019 (3), 2020 (4)	AMP (6), PEN (6), FOX (9), GEN (4), ERY (5), TET (2)
CC72	0/2	ST72 (2)	Stool (2)	2019 (1), 2021 (1)	PEN (2)
CC88	1/0	ST88 (1)	Stool (1)	2014 (1)	AMP (1), FOX (1), PEN (2)
CC188	0/9	ST188 (9)	Stool (9)	2011 (2),2019 (6), 2020 (1)	AMP (8), PEN (8), ERY (1)
CC398	8/17	ST398 (25)	Stool (25)	2011 (2),2016 (1), 2019 (10), 2020 (6), 2021 (6)	AMP (18), PEN (18), FOX (8), ERY (6)
CC1281	0/10	ST1281 (10)	Stool (9), Vomit (1)	2011 (3), 2014 (1), 2019 (4), 2020 (1), 2021 (1)	AMP (2), ERY (2), FOX (1), GEN (2), PEN (2), TET (1)
ST672	0/1	ST672 (1)	Stool (1)	2020 (1)	PEN (1)
ST944	0/1	ST944 (1)	Stool (1)	2019 (1)	PEN (1)
ST1920	0/1	ST1920 (1)	Stool (1)	2019 (1)	PEN (1), GEN (1)
ST2315	0/2	ST2315 (2)	Stool (2)	2019 (1), 2020 (1)	AMP (2), PEN (2), ERY (1), TET (1), GEN (1)
ND	1/2	Unknown (n = 3)	Stool (3)	2011 (1), 2019 (1), 2021 (1)	PEN (1), FOX (1), ERY (1)

a Numbers in parentheses are the number of isolates per sequence (ST) type.

b Numbers in parentheses are the number of isolates. ND: not determined.

c Numbers in parentheses are the number of isolates resistant to the antimicrobial.

**Figure 3:**
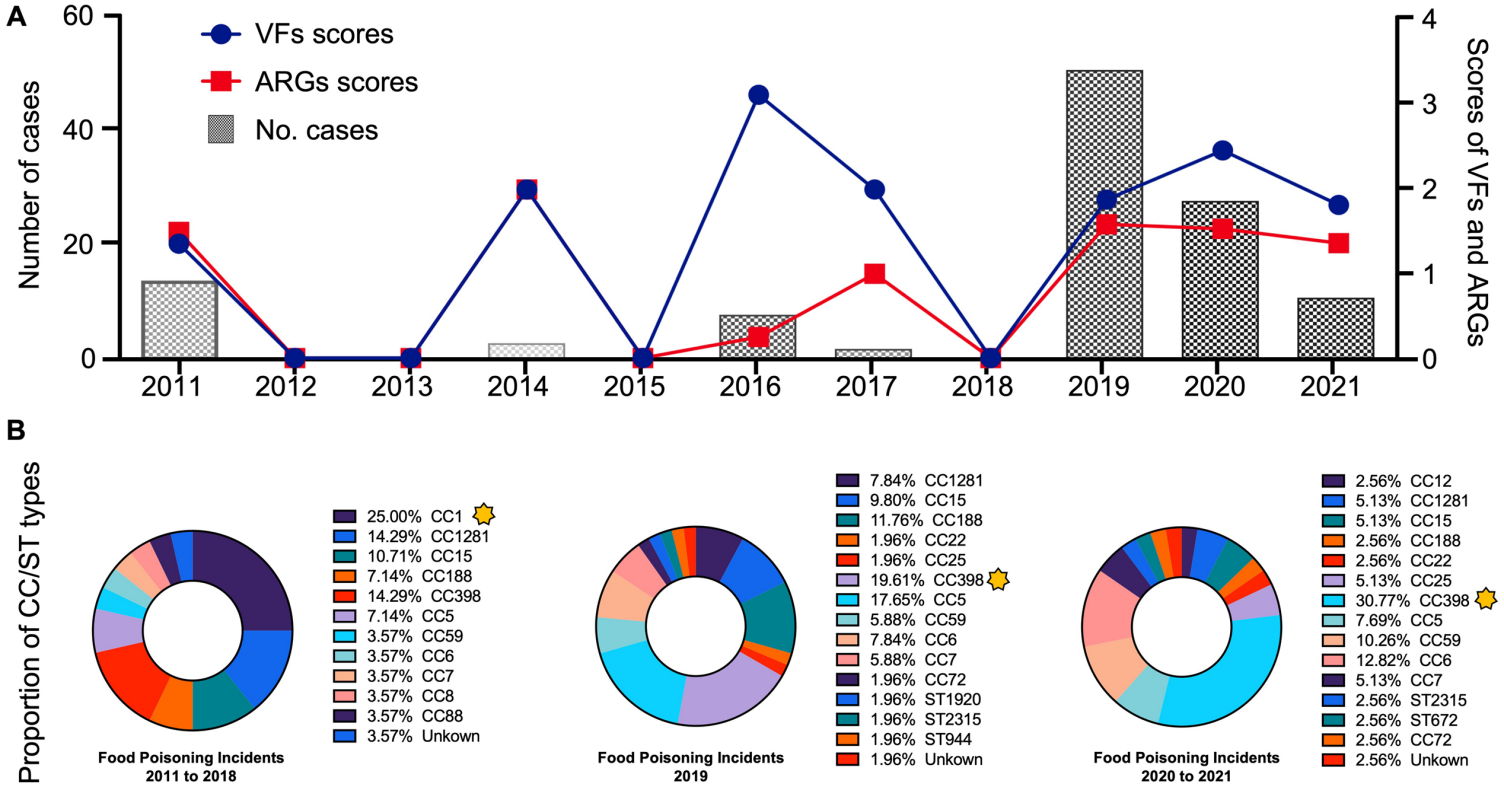
**(A)** The line chart represented the total number of ARGs and VFs from 117 *S. aureus* strains between 2011 to 2021. **(B)** The donut chart represented the proportion of CC and ST types of *S. aureus* strains in the current food poisoning incidents. The yellow stars marked the dominant types, which showed a considerable shift in CC types from CC1 to CC398 between 2011 and 2021.

An intriguing finding from this study was the considerable shift in CC types from CC1 to CC398 between 2011 and 2021 (Figure 3B). Before 2018, food poisoning incidents were primarily linked to community-associated CC1. However, the subsequent five years witnessed an increase in the CC398 strains associated with livestock settings, indicating the intricate nature of foodborne *S. aureus* outbreaks.

Region IV and Region II were identified as the most prevalent regions among the current food poisoning incidents, with CC398 (n = 25), CC5 (n = 14), CC6 (n = 10), and CC15 (n = 10) being the dominant types (Figure 2). Historically, *S. aureus* CC8, CC15, and CC45 had been the predominant strains causing foodborne diarrhea in European countries (Baumgartner et al., 2014). Analysis of 1850 food products from China revealed that *S. aureus* CC1 (10.7%) was the most common type, followed by CC7 (10.6%) and CC5 (4.8%), suggesting a different major variation compared to the food poisoning incidents we found in this study (Wu et al., 2018). This disparity could potentially be attributed to differences in Chinese and Western food cultures, as well as variations in cooking practices.

CC398 was identified as the most predominant clonal complex in this study (Figure 2, Table 1). Previous retrospective analysis revealed that human and animal infections with *S. aureus* CC398 occurred in nations throughout West Europe and Eastern Asia, with the initial cases harboring the strain being identified in the Netherlands (Stegger et al., 2010; Laumay et al., 2021). Since then, the annual growth in the detection rate of CC398 had been increasing, reaching 20.0% in 2006 in Europe (Laumay et al., 2021). In China, the positive rate for CC398 strains in livestock-associated food products varied between 4.6 to 33.3% depending on the region (Wu et al., 2018; Li W. et al., 2021). Similarly, CC398 was detected in meat products with a prevalence of 11 to 31%, indicating that CC398 had entered the food chain and performed as the primary epidemic strain circulating over the world (Verkade and Kluytmans, 2014).

*S. aureus* CC5 was a common community-associated MRSA lineage circulating in poultry (Aires-de-Sousa, 2017). Studies identified that the majority of *S. aureus* isolated from poultry belonged to the avian-associated spectrum of CC5, which emerged from a human-to-poultry host jump and was characterized by numerous signatures of adaptation to the avian host including carriage of the ΦAVβ prophage genes (Matuszewska et al., 2020). In the present study, a local blast was performed to detect the ΦAVβ genes (SAAV_2008, SAAV_2009) in *S. aureus* strains (Lowder et al., 2009; Tang et al., 2017). All CC5 isolates carried ΦAVβ genes, which was consistent with the identification of these isolates in poultry products from current food poisoning incidents (Figure 2, Table 1).

**Figure 4:**
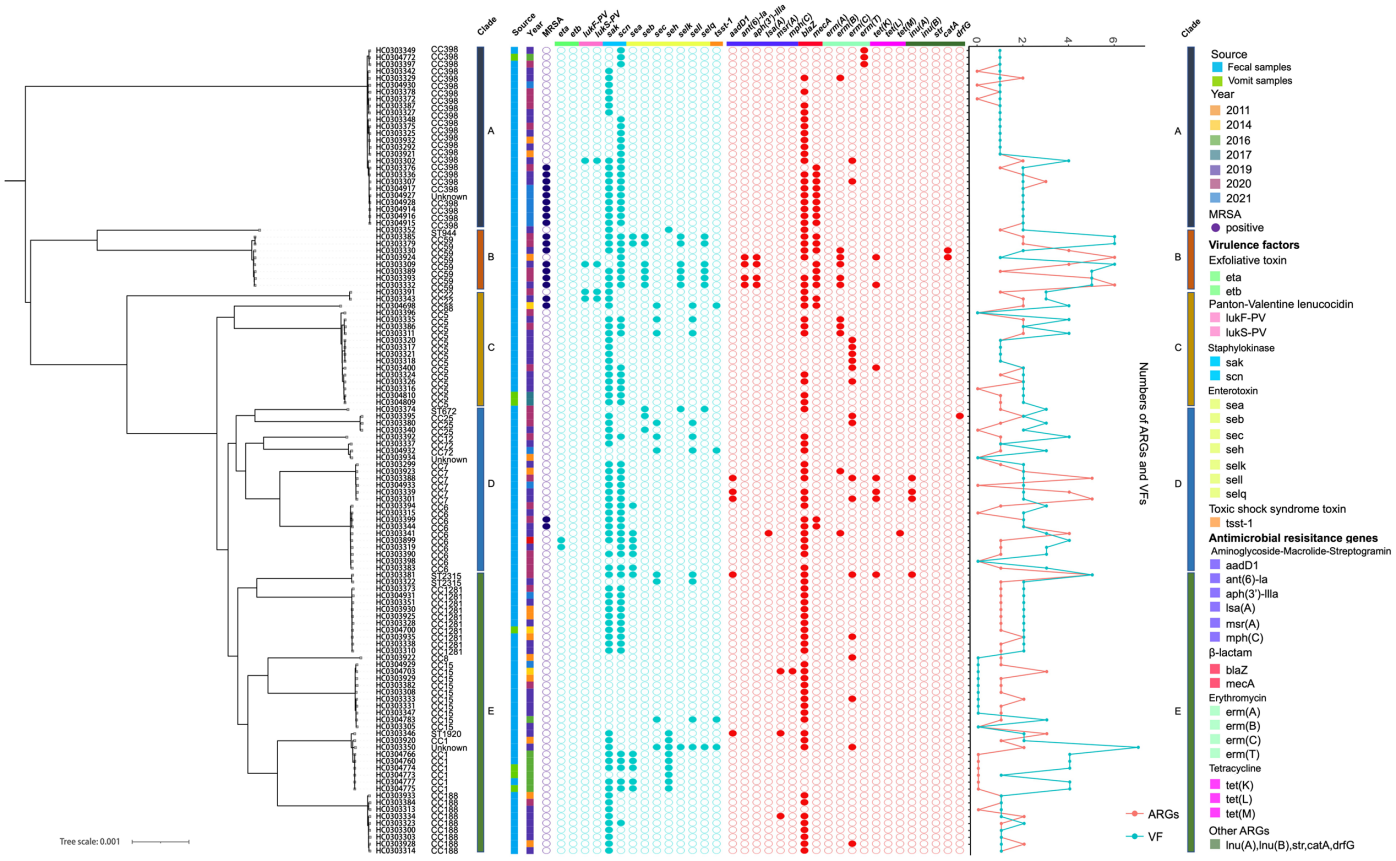
Genetic characteristics of 117 foodborne *S. aureus* strains. The clade, source, year, antimicrobial and virulence genes are depicted by colored squares. Filled or empty circles indicate the presence (filled) or absence (empty) of MRSA (violet), antimicrobial (red) and virulence (turquoise) genes in the 117 foodborne *S. aureus* strains. The line graph represents the total number of ARGs and VFs in the 117 strains.

According to previous studies, CC6 was identified as a prevalent pathogen causing food poisoning in adults. Previous study indicated that a total of 868 *S. aureus* isolates were collected from meat products in China, of which 47 strains were CC6, accounting for about 5.4% (Wu et al., 2018). There was evidence that *S. aureus* CC6 variations were isolated from diarrhea, and virulence varied between enterotoxin-encoding mobile genetic elements in Eastern Asia (Suzuki et al., 2014). In this study, CC6 isolates carried antimicrobial resistance genes such as *aadD1, blaZ, mecA, ermB,* and *tetM*, indicating a multidrug-resistant (MDR) spectrum in China (Figure 4, Table 1). As a result, it is imperative to develop tools for accurate screening for MDR *S. aureus* in foods.

The final predominant strain that caused diarrhea in food poisoning incidents was *S. aureus* CC15. Previous studies reported that a total of 8 CC types and 12 ST types of *S. aureus* were isolated from food poisoning incidents, with CC15 accounting for 5% of the total and carrying numerous enterotoxin genes such as *sec*, *sed*, and *see* (Lv et al., 2021). However, CC15 isolated in this study was not found to carry any enterotoxin genes, nor as *luk*F-PV and *luk*S-PV, suggesting a low pathogenicity but a high prevalence of hypotonic *S. aureus* contamination in food poisoning incidents in Eastern China (Figure 4, Table 1).

### Virulence factors of *Staphylococcus aureus* isolates from the food poisoning incidents

One of the significant causes of diarrhea and other gastrointestinal problems was *S. aureus* enterotoxin genes (SEs). Genes for enterotoxins were found in 55 isolates in this investigation, with a prevalence of 27.8% (27/97) for MSSA and 3.5% (7/20) for MRSA (Table 2). CC1, CC5, CC25, and CC59 commonly had part of the *sea, seb, sec, seh, selk, sell,* and *selq* genes, however, neither clade A nor clade D contained any enterotoxin genes, except for five *sea* genes present in CC6 (Figure 4, Table 2).

**Table 2:** Distribution of antimicrobial resistance genes according to sensitivity toward methicillin (MSSA vs. MRSA) and CC types.

	No.^a^	Aminoglycoside genes^b^	Beta-lactam genes		Macrolide, Lincosamide, and Streptogramin B genes^f^	Tetracycline genes^g^	Trimethoprim genes^h^	Chloramphenicol genes^i^	Multi-drug resistance (%)
			Penicillin^c^	Methicillin^d^					
Methicillin sensitivity									
MSSA	97	7	68	0	35	7	1	1	5 (5.2%)
MRSA	20	5	17	14	5	1	0	1	5 (25.0%)
CC types									
CC398	25	0	18	8	6	0	0	0	0
CC5	14	0	7	0	9	1	0	0	0
CC6	10	0	8	2	1	1	0	0	0
CC15	10	0	9	0	3	0	0	0	0

a Numbers of certain *S. aureus* strains.

b ant(6)-Ia-like, aph(3′)-III, aadD-like, and str genes.

c blaZ gene.

d mecA gene. eqnrB19 gene.

f erm(A), erm(B), erm(C), erm(T), vga(E), lnu(A), lnu(B), mph(C), and msr(A) genes.

g tet(K), tet(L), and tet(M) genes.

h dfrA1, dfrK, and dfrG genes.

i catA1 gene.

The carrier rate of foodborne *S. aureus* enterotoxins varied greatly. A study examining the rate of classical *staphylococcus* enterotoxins revealed that 39.3% of the foodborne isolates were enterotoxin-positive, with *sec* and *sea* as the dominant genes (Zhang et al., 2022). Moreover, a correlation was observed between the prevalence of specific enterotoxin genes, e.g., *sea* and *seh*, with the severity of the associated illness (Argudín et al., 2010). This finding underscores the importance of monitoring the prevalence of enterotoxins in foodborne *S. aureus* and its relevance to outbreak tracing.

The pathogenicity of these *S. aureus* isolates differed from that of other virulent factors. A total of 14 virulent factors, including the functional components Panton-Valentine leucocidin, toxic shock syndrome toxin, staphylokinase, exfoliative toxin, and enterotoxin, were examined among 117 foodborne *S. aureus* strains. Of the 117 isolates, only two exfoliative toxin-positive genes (*eta*) and four *tsst*-1-positive genes were detected, showing that *S. aureus* isolates from the foodborne epidemic was hypotonic and enterotoxins were the predominant pathogenicity in the context of food safety (Figure 4, Table 2).

The *pvl* gene was detected only in four isolates (4/117, 3.4%) in this study, which was surpassed by prior research that found Panton-Valentine leucocidin to be detectable in MRSA at a rate of 24.1% (Wu et al., 2019). Such a high PVL carriage rate among MRSA isolates was observed for food in China, suggesting considerable diversity in the frequency of PVL across foodborne *S. aureus* strains.

Furthermore, a total of 69 isolates were confirmed to contain *scn* genes, including 18 isolates from the MRSA group (Figure 4, Table 2). As a recognized marker of the immune evasion cluster (IEC), the *scn* gene was found in high frequency in human hosts, indicating that the gene may be utilized to differentiate strains that are transferred to humans from environments and animals (de Jong et al., 2018). The *scn* gene appeared to be more prevalent in the MSSA group. An earlier study conducted in China found an 81.3% (52/64) prevalence of *scn* in MSSA isolates from the intestinal tracts of adult patients (Li et al., 2022). In our study, the *scn* gene was detected in more than 59.0% of the isolates, demonstrating that the causes of infections in food poisoning incidents were widespread, and included both human-to-human transmission and environmental and livestock-associated contaminations.

Genes associated with the biofilm formation were predicted in the *S. aureus* strains. Interestingly, the *icaC, icaD, icaR*, and capsule genes (*capA* to *capP*) were found in majority of the 117 isolates, whereas *icaB* was found in 107 strains (Supplementary Table S4, Supplementary Figure S1). Furthermore, *arg* and *luxS* genes were found in all isolates, which were linked to pathogenicity, such as Quorum Sensing, demonstrating the potential ability to form biofilm in connection to pathogenicity in *S. aureus* (Reading and Sperandio, 2006) (see Table 3).

**Table 3:** Distribution of virulence factors according to sensitivity toward methicillin (MSSA vs. MRSA) and CC types.

	No	Enterotoxins													Exfoliative toxins		Toxic shock syndrome	Other virulence factors		SEs positive^a^
		*sea/sep*	*seb*	*sec*	*seg*	*seh*	*sei*	*sek*	*sel*	*sem*	*sen*	*seo*	*seq*	*seu*	*eta*	*etb*	*tst*	*luk*F-PV	*scn*	(%)
Methicillin sensitivity																				
MSSA	97	12	3	9	0	10	0	2	9	0	0	0	2	0	2	0	3	2	51	27 (27.8%)
MRSA	20	2	6	1	0	0	0	6	1	0	0	0	6	0	0	0	1	2	18	7 (35.0%)
CC types																				
CC398	25	0	0	0	0	0	0	0	0	0	0	0	0	0	0	0	0	1	18	0 (0.0%)
CC5	14	0	0	2	0	0	0	0	2	0	0	0	0	0	0	0	0	0	9	2 (14.3%)
CC6	10	6	0	0	0	0	0	0	0	0	0	0	0	0	2	0	0	0	9	6 (60.0%)
CC15	10	0	0	1	0	0	0	0	1	0	0	0	0	0	0	0	1	0	0	1 (10.0%)

a Numbers in parentheses indicate the prevalence of enterotoxin genes in each category.

Finally, prophage ΦSa3 and ΦAVβ were identified in this study (Supplementary Table S4, Supplementary Figure S1). The majority of human-associated *S. aureus* harbored β-hemolysin negative-converting bacteriophages, which are classified as ΦSa3 (Goerke et al., 2009). In this study, prophage ΦSa3 was found in 69 isolates, which was consistent with the findings of the *scn* genes and indicated that *S. aureus* has jumped its host from animals to humans. Additionally, ΦAVβ prophage genes were the indicators of the human-to-poultry host jump (Lowder et al., 2009). A total of 14 isolates were found to harbor the ΦAVβ genes (SAAV_2008, SAAV_2009) in CC5 strains, which showed that chicken items may have been a source of contamination for the current food poisoning incidents. However, further research is required to elucidate the bacteriophages of pathogenic *S. aureus* in food poisoning incidents.

### Antimicrobial resistance of *Staphylococcus aureus* isolates from food poisoning incidents

The antimicrobial susceptibility pattern observed in *S. aureus* isolates from various outbreaks revealed that only ten isolates displayed resistance to at least one antimicrobial, including penicillin, tetracycline, and macrolides (Figure 4, Table 2, Supplementary Table S5). Antimicrobial resistance indicators were substantially more prevalent in CC398 isolates than in other CC types. Macrolide, Lincosamide, and Streptogramin B genes were found in 36.8% of MSSA isolates and 25.0% of MRSA isolates, respectively. Similar to previous research, we found that 93.0% of the *S. aureus* isolates in our investigation were penicillin-resistant (Table 2) (Li et al., 2022).

The frequency of antimicrobial resistance genes from *S. aureus* in diarrhea patients had been extensively investigated. For instance, a total of 187 *S. aureus* clinical isolates were collected in South China from 2010 to 2016 (Liang et al., 2019). Among them, 103 isolates were identified as MRSA with resistance to erythromycin (64.1%), clindamycin (48.5%), gentamicin (36.9%) and ciprofloxacin (34.0%). These findings demonstrated that the therapeutic management of hypervirulent MRSA infection may be complicated by MDR isolates colonizing livestock.

Intriguingly, tetracycline resistance is a hallmark of the CC398 clade, and tetracycline accumulation in CC398 has been reported to be associated with rapid radiation from humans to livestock (Price et al., 2012). However, all the CC398 strains were tetracycline susceptible in this study. This may be explained by the fact that the current strains were all isolated from human infection instead of livestock origination. In a prior study, we conducted a sampling of pork in Beijing and found that the livestock-associated CC398 harbored a high number of tetracycline resistance genes including *tetK*, and *tetM* (Li H. et al., 2021). Altogether, our findings support the differential carriage of tetracycline resistance in *S. aureus* between humans and livestock reported by Price et al.

Furthermore, CC59 was found to harbor a bunch of antimicrobial resistance and virulence genes including *eta, etb, scn, sea,seb,selk, selq, ant(6)-la, blaZ, erm(B), tet(K)* and others in this study. CC59 was a predominant clonal lineage of community-acquired *S. aureus* circulating in Asia (Pang et al., 2020). Previous research described two distinct clones of the ST59 sequence type, PVL-negative/SAK-positive and PVL-positive/SAK-negative (Hung et al., 2016). In this study, a similar mode of distinct clones was observed that seven CC59 strains were PVL-negative/SAK-positive and the rest one was PVL-positive/SAK-negative. Moreover, CC59 isolated from food chain were reported to be resistant to ampicillin, penicillin, erythromycin, tetracycline and others (Pang et al., 2020), which was consistent with the results in this study.

In addition, vancomycin-resistant *S. aureus* strains were reportedly isolated from food products in Egypt, with a detection rate of 64.7% for *vanA* and 29.4% for *vanB* (Saber et al., 2022). However, none of the 117 foodborne *S. aureus* isolates in this study tested positive for *vanA* (Supplementary Table S3). The *S. aureus* virulence factors linked to staphylococcal food poisoning are of importance in terms of food safety. In this study, the enterotoxin A, B, and C genes (*sea*/*seb*/*s*) were shown to be the most frequent enterotoxin genes in the current foodborne *S. aureus* isolates. These enterotoxins have been linked to staphylococcal food poisoning as well as toxic shock syndrome in humans (Benkerroum, 2018). Although our results suggested that these diarrhea episodes were hypotonic and merely transient low-MDR infections, however, further research for continued surveillance given the detection of virulence and antimicrobial resistance determinants is required to elucidate the genomic characteristics of pathogenic *S. aureus* in food poisoning incidents in the context of public health.

This study has some limitations. This investigation primarily focused on the events produced by *S. aureus* due to the range of pathogenic bacteria leading to food poisoning incidents. Nevertheless, diarrhea caused by *Salmonella* and *Shigella* was more frequent in most cases (Kotloff, 2022). Additionally, the sample size and strain counts were limited and only covered the period from 2011 to 2021 in terms of food poisoning incidents in Eastern China. To enhance the utilization of genetics in food safety research and manufacturing, it is crucial to gather additional data on foodborne *S. aureus* in future studies.

## Conclusion

In conclusion, a combined bioinformatics approach was employed to study the genetic characteristics of *S. aureus* strains isolated from food poisoning incidents in Eastern China from 2011 to 2021. A number of 19 unique CC/ST types were identified among the foodborne *S. aureus* genomes, with CC398, CC5, CC6, and CC15 being the dominant types, respectively. Genes for enterotoxins were found in 55 isolates, while the rest virulence factors were merely detected, showing that *S. aureus* isolates from the foodborne epidemic were hypotonic and enterotoxins were the predominant pathogenicity. Antimicrobial resistance indicators were substantially more prevalent in CC398 isolates, however, only ten isolates displayed multi-drug resistance in the present study, suggesting that these diarrhea episodes may not pose a major clinical risk for treatment-resistance infections among the food poisoning incidents that occurred in Eastern China between 2011 to 2021.

## Data availability statement

The datasets presented in this study can be found in online repositories. The names of the repository/repositories and accession number(s) can be found below: CRA010922 (https://ngdc.cncb.ac.cn/gsa/browse/CRA010922).

## Ethics statement

The studies involving humans were approved by Research Ethics Committee of Soochow University. The studies were conducted in accordance with the local legislation and institutional requirements. The participants provided their written informed consent to participate in this study.

## Author contributions

SY, YZ, DF, QJ, and TL performed the analysis. HL and ZZ wrote the main manuscript text and GJ prepared the figures. All authors contributed to the article and approved the submitted version.

## Funding

The project was supported by the National Natural Science Foundation of China (No. 32170003 to ZZ, No. 82202465 to HL). This work was supported by agricultural innovation grants from Suzhou Science and Technology Project (N316460121 to HL) and college students’ innovation and entrepreneurship training program of Soochow University (202310285177Y to SY).

## Conflict of interest

The authors declare that the research was conducted in the absence of any commercial or financial relationships that could be construed as a potential conflict of interest.

## Publisher’s note

All claims expressed in this article are solely those of the authors and do not necessarily represent those of their affiliated organizations, or those of the publisher, the editors and the reviewers. Any product that may be evaluated in this article, or claim that may be made by its manufacturer, is not guaranteed or endorsed by the publisher.

## Footnotes

^1^http://www.genomicepidemiology.org
